# Microbial signature in IgE-mediated food allergies

**DOI:** 10.1186/s13073-020-00789-4

**Published:** 2020-10-27

**Authors:** Michael R. Goldberg, Hadar Mor, Dafna Magid Neriya, Faiga Magzal, Efrat Muller, Michael Y. Appel, Liat Nachshon, Elhanan Borenstein, Snait Tamir, Yoram Louzoun, Ilan Youngster, Arnon Elizur, Omry Koren

**Affiliations:** 1 Yitzhak Shamir Medical Center (Assaf Harofeh), Zerifin, Israel; 2 Department of Pediatrics, Sackler Faculty of Medicine, Tel Aviv, Israel; 3 The Azrieli Faculty of Medicine, Bar Ilan University, Safed, Israel; 4 The Mina and Everard Goodman Faculty of Life Sciences, Bar-Ilan University, Ramat-Gan, Israel; 5 MIGAL-Galilee Research Institute, Kiryat Shmona, Israel; 6 Tel-Hai College, Upper Galilee, Israel; 7 The Blavatnik School of Computer Science, Tel Aviv University, Tel Aviv, Israel; 8 Department of Medicine, Sackler Faculty of Medicine, Tel Aviv University, Tel Aviv, Israel; 9 Department of Clinical Microbiology and Immunology, Sackler Faculty of Medicine, Tel Aviv University, Tel Aviv, Israel; 10 Santa Fe Institute, Santa Fe, NM USA; 11 Department of Mathematics, Bar-Ilan University, Ramat-Gan, Israel

**Keywords:** Food allergy, Microbiota, *P*. *copri*, SCFA, Prebiotics, Postbiotics, Supervised learning

## Abstract

**Background:**

Multiple studies suggest a key role for gut microbiota in IgE-mediated food allergy (FA) development, but to date, none has studied it in the persistent state.

**Methods:**

To characterize the gut microbiota composition and short-chain fatty acid (SCFAs) profiles associated with major food allergy groups, we recruited 233 patients with FA including milk (*N* = 66), sesame (*N* = 38), peanut (*N* = 71), and tree nuts (*N* = 58), and non-allergic controls (*N* = 58). DNA was isolated from fecal samples, and 16S rRNA gene sequences were analyzed. SCFAs in stool were analyzed from patients with a single allergy (*N* = 84) and controls (*N* = 31).

**Results:**

The gut microbiota composition of allergic patients was significantly different compared to age-matched controls both in α-diversity and β-diversity. Distinct microbial signatures were noted for FA to different foods. *Prevotella copri* (*P. copri*) was the most overrepresented species in non-allergic controls. SCFAs levels were significantly higher in the non-allergic compared to the FA groups, whereas *P. copri* significantly correlated with all three SCFAs. We used these microbial differences to distinguish between FA patients and non-allergic healthy controls with an area under the curve of 0.90, and for the classification of FA patients according to their FA types using a supervised learning algorithm. *Bacteroides* and *P. copri* were identified as taxa potentially contributing to KEGG acetate-related pathways enriched in non-allergic compared to FA. In addition, overall pathway dissimilarities were found among different FAs.

**Conclusions:**

Our results demonstrate a link between IgE-mediated FA and the composition and metabolic activity of the gut microbiota.

## Background

Food allergy (FA) is defined as an adverse health effect arising from an IgE-mediated immune response that occurs reproducibly upon exposure to specific food antigens. The prevalence of allergic disorders has been increasing worldwide, and FA is of particular concern considering the severity of its clinical manifestations and the persistence of the disorder past childhood. While a majority of egg and milk allergies resolve by age four [1], a significant minority persist into adulthood. In addition, the majority of patients who suffer from peanut and tree nuts allergies tend to persist into adulthood. It is estimated that FA affects up to 8% of children and 3% of adults in industrialized countries [2].

Mechanistically, FA emerges in the context of oral tolerance failure. The establishment and maintenance of oral tolerance depend on the interplay between several biological systems, most notably the immune system, the intestinal epithelium, and the gut microbiome. The latter component promotes the maintenance of the intestinal barrier and modulates the function of resident immune cells through the generation of metabolites such as short-chain fatty acids (SCFAs) [3, 4]. For example, in germ-free (GF) mice who do not produce SCFAs as they lack a gut microbiota, treatment with acetate markedly improved disease indices and decreased levels of inflammatory mediators, in a colitis model [4]. Additionally, several studies conducted in mice suggested that SCFAs may play a role in allergy protection, including protection against allergic airway diseases and decrease of allergic airway inflammation [4–6]. It was also reported that a high-fiber diet could promote oral tolerance and confer protection against FA in mice via SCFA signaling through several pathways, such as G-protein-coupled receptor GPR43 signaling [4, 7], mediated by acetate and propionate [8, 9]. On a cellular level, SCFAs act to promote the development of tolerogenic CD103^+^ dendritic cells, which influence the development of regulatory T cells [10] and drive B cell production of IgA [11]. Conversely, immune dysregulation may result from changes in the gut microbiota composition [12] leading to changes in the above cellular milieu, and in the case of allergy leading to production of Th2 cytokines and food allergen-specific IgE [13].

Previous work has indicated potential links between microbiota composition and the development of FA. Utilizing a mouse model of fecal transplantation from specific pathogen-free (SPF) to germ-free (GF) mice, a causative role for the commensal bacteria in protecting against food sensitization was demonstrated [14]. Clostridia-containing microbiota induced innate lymphoid cells to significantly increase IL-22 production which reinforced the epithelial barrier to reduce intestinal permeability to dietary antigens limiting allergen access to the bloodstream. Several human studies examined early-life microbiota composition in relation to eventual FA outcome [15, 16]. Other studies compared microbiota between allergic and tolerant children for FA generally [17, 18] and for specific allergies [19]. Despite the reported features distinguishing allergic microbiota, few studies with a large number of participants have examined the microbiota in FA across multiple allergies in the persistent state. This work presents a large-scale study of microbiota association with FA in over 200 patients with a variety of allergies. Herein, we demonstrate that there is a bold microbial signature concomitant with significantly lower concentrations of stool SCFAs in FA patients.

## Methods

### Study cohort

To characterize the gut microbiota composition and short-chain fatty acids (SCFAs) profiles associated with major food allergy groups, we prospectively enrolled 233 allergic patients (> 4 years old) from a single center (Institute of Allergy, Immunology and Pediatric Pulmonology, Shamir Medical Center) referred for evaluation of IgE-mediated food allergy. Patients were allergic to milk (*N* = 66, 58 singly allergic), peanut (*N* = 71, 44 single), sesame (*N* = 38, 12 single), or tree nuts (*N* = 58, 52 single). Four additional egg-allergic patients were initially recruited, but not included in the analysis, due to their small sample size. Inclusion criteria were patients with an oral food challenge confirmed food allergy or history of a reaction in the past year in the presence of IgE-reactivity as evidenced by a positive skin prick test and an age > 48 months. Stable asthmatics were eligible for inclusion. Exclusion criteria included oral antibiotic use in the prior 3 months and a history of other numerous chronic inflammatory diseases, including inflammatory bowel disease and the group of obesity-associated diseases collectively referred to as metabolic syndrome. A detailed medical history including the presence of other allergic comorbidities (other food allergies, atopic dermatitis, and asthma) was obtained. Patients were skin prick tested (SPT) to suspected allergens and oral food challenges (OFC) were performed. High concentration SPT were utilized for sesame [20] and tree nuts [21] as previously described and commercial reagents for milk and peanut (1:10 w/v, ALK-Abello, Port Washington, NY, USA) with histamine being used as a positive control (1 mg/mL; ALK-Abello, Port Washington, NY, USA). OFC were done according to previously published protocols [1, 22–24] reaching maximum doses for milk, peanut, sesame, and tree nuts of 3600, 3000, 4080, and 4000 mg protein, respectively. The diagnosis of allergy was based on a recent reaction (cutaneous, gastrointestinal, respiratory and/or systemic symptoms occurring within 2 h of exposure) or a positive OFC, together with evidence of sIgE reactivity on SPT. Patients were categorized as tolerant to a specific allergen if they reported regular consumption of that food or if they had a negative OFC. Stool samples were collected from FA patients prior to challenge and from healthy non-allergic age-matched controls (Bar Ilan University) and stored at − 80 °C until use. The study was approved by the Institutional Review Board (#0022-16-ASF) and conformed to the principles of the Helsinki Declaration, and all patients or their caretakers signed full informed consent. The control samples (*N* = 58) were collected under the approval of Ethics in Research (human subjects) Committee at the Azrieli Faculty of Medicine, Bar-Ilan University (approval #112015). All samples were processed together in order to avoid batch effects.

### DNA extraction and 16S rRNA gene amplification

DNA was extracted from all fecal samples using the Mobio PowerSoil DNA extraction kit, as described by the manufacturer (MoBio, Carlsbad, CA). The samples were homogenized using a beadbeater for 2 min. Bacterial 16S rRNA gene sequences from each sample were amplified by PCR performed in 96-well plates. Sample preparation was similar to that described by Nuriel-Ohayon et al. [25]. Briefly, PCR reactions contained 17 μL PCR grade nuclease-free water (Hylabs, Rehovot, Israel), 25 μL PrimeSTAR Max (Takara-Clontech, Shiga, Japan), 2 μL each of the forward and reverse primers (10 μM final concentration), and 4 μL genomic DNA. Reactions were heated to 95 °C for 3 min for DNA denaturation, with amplification proceeding for 33 cycles at 98 °C for 10 min, 55 °C for 5 min, and 72 °C for 5 min; a final extension of 1 min at 72 °C was added to ensure complete amplification. Barcoded universal primers 515F and 806R containing Illumina adapter sequences which target the highly conserved V4 region, as previously described [25], were used to amplify the microbiota from individual samples. Amplicons were purified using AMPure XP magnetic beads (Beckman Coulter, Brea, CA) and subsequently quantified using Quant-it Picogreen dsDNA quantification kit (Invitrogen, Carlsbad, CA). Equimolar ratios of amplicons from individual samples were pooled together before sequencing on the Illumina MiSeq platform at the Genomic Center of the Bar Ilan University, at the Azrieli Faculty of Medicine.

### Data analyses

Microbial communities were analyzed using QIIME2 version 2019.4 [26]. Sequences were quality filtered to remove short and long sequences, sequences with primer mismatches, uncorrectable barcodes, and ambiguous bases. Read normalization was performed before the analysis output with rarefaction of 11,500 sequences per sample and features with less than 0.0003% of total reads were discarded. A closed reference Greengenes database [27] was used to pick taxonomic features and assign taxonomy. ANCOM [28] and Python (version 3.7, library Seaborn) platforms were used to identify significantly differentiating microbial taxa among groups. Sequences are available in the European Nucleotide Archive repository with the accession number PRJEB37877.

### SCFA extraction and analysis

An aliquot of 0.25 g of wet feces was thawed and suspended in 1 mL of an orthophosphoric acid solution (8% v/v) and kept at room temperature for 10 min with occasional shaking. The mixture was homogenized for 2 min, and the suspension was centrifuged at 4 °C for 15 min at 14,000 rpm. The supernatant was filtered by centrifugation at 4 °C for 15 min at 14,000 rpm. Next, 225 μL of the supernatant was transferred into a polypropylene tube, and 25 μL of 2-methyl-butyric-acid (Sigma-Aldrich, USA) was added as an internal standard (IS) to a final concentration of 0.001 M and transferred to a chromatographic vial for gas chromatography analyses. The IS was used to correct for injection variability between samples and minor changes in the instrument response. Vials were stored at − 20 °C before GC analysis. A standard mix (WSFA-4, Sigma-Aldrich, USA) was used to determine the concentrations of propionic acid. Standard curves for acetic acid and butyric acid (Sigma-Aldrich, USA) were prepared using stock solutions of both acids, separately. Chromatographic analyses were carried out using the Agilent Technologies 6890 A GC system with a mass selective detector. Fused-silica capillary column with a free fatty acid phase (DB-FFAP 122-3232, 30 m × 0.25 mm × 0.25 μm) was used. The carrier gas was helium at the flow rate equal to 13.6 mL/min. The initial oven temperature was 70 °C, raised to 100 °C at 20 °C/min, then raised to 180 °C at 8 °C/min and held for 3 min, and then raised to 230 °C at 20 °C/min. The injection volume was 1 μL and the run time of a single analysis was 17 min.

### Statistical analysis of the 16S rRNA, SCFAs data, and metadata

We employed Spearman rank correlations for the continuous metadata and ANOVA for the categorical metadata to check for associations between the metadata obtained (FA, SCFAs concentrations, demographic and clinical data) and the abundances of the microbial features summarized at different taxonomic levels. For the analysis involving multiple comparisons, we performed pairwise *t* test on the bacterial relative abundances and the SCFA concentrations and adjusted using false discovery rate (FDR). Statistical analyses were performed using R version 1.1.463.

To identify correlations between microbial taxa and SCFAs, we calculated the Spearman correlation between the normalized log bacterial expression level and the SCFAs and performed a two way (row and column) single link Euclidean hierarchical clustering. Python’s Seaborn library was used for statistics of Spearman rank correlations and visualization of all the represented heatmap plots.

### Functional profile prediction and identification of taxonomic drivers

PICRUSt2 [29] was used to predict KO abundance profiles from taxonomic profiles. FishTaco platform [30] was used to identify KEGG pathways that are differentially abundant between microbiomes of different study groups and the taxa that are driving these functional shifts. Briefly, FishTaco receives as input the taxonomic profiles obtained by QIIME2, a genomic content table, and the KO abundance profiles as generated by PICRUSt2 [29], calculates the differential abundance score for each pathway, and uses a permutation and Shapley-values-based method to decompose observed functional shifts into taxon-based contributions.

### Machine learning (ML)

Features were aggregated to the genus level by averaging over all features assigned to the same genus. Given the large variation in feature values, we transformed these values to *Z* scores by adding a minimal value to each feature level (0.01) and calculating the 10-basis log of each value. Statistical Whitening was then performed on the table, by removing the average and dividing by the standard deviation of each sample. Supervised Learning was performed on the normalized and aggregated version of the 16S rRNA feature table, including all the taxonomical data, in order to recognize patterns in the data. Principal Component Analysis was performed using Python version 3.7 and its package and sklearn library. The first 20 components were kept. These components explain 60.24% of the variance. A binary linear Support Vector Machine was trained to classify healthy non-allergic donors and allergic patients based on all bacterial abundance features. Five-fold cross-validation was performed. The box constraint value was 1 and weighted by the group size. More complex methods were not used to limit overfitting, given the limited number of samples. The SVM score was used to produce a receiver operating characteristic (ROC) curve on the test set. The reported area under curve (AUC) is the average AUC over all test sets. For the multi-class analysis, a multi-class weighted linear SVM was trained, with a similar configuration and box constraint value of 0.1. The error was estimated through a confusion matrix representing the relative fraction of cases in the test set where class *i* was predicted while the truth was class *j*. The accuracy was defined as the sum of the diagonal.

## Results

To study the associations between FA and patient’s clinical data and the abundances of the microbial taxa, we used 16S rRNA gene sequencing and computational methods. We characterized features of the gut microbiota in fecal samples, identified microbial patterns associated with FA in a cohort of 233 patients with various types of FA, and compared the microbiota with 58 samples from healthy controls. The demographic features of the study participants are summarized in Table 1.

**Table 1:** Demographic characteristics of the study cohort

Parameter	Control (***n*** = 58)	Allergic (***n*** = 233)	***p*** value
**Age, months (IQR)**	78 (48.0–125.3)	77 (63.0–114.5)	NS
**Gender, M (%)**	56.9	59.5	NS
**BMI** ***z*-score median (IQR)**	0.6 (− 0.73–1.39)	0.13 (− 0.54–0.805)	NS

To discard possible covariates affecting the microbiome, we examined the influence of the demographic and clinical characteristics on the microbiome in the allergic group. We tested various groupings of allergic patients according to different personal or clinical characteristics, e.g., gender, age, asthma, eosinophil blood level, and adrenaline requirement. We found no significant differences in the α or β diversity between any of the comparisons we tested. Moreover, the *R*^2^ for the beta diversity (Weighted UniFrac) was zero for most of the comparisons except for age (*R*^2^ = 0.006) and extreme levels of eosinophils (*R*^2^ = 0.001), meaning only 0–0.6% of the variances we examined could be explained by the microbiome (Table 2). In addition, neither ANCOM nor spearman correlations identified any bacterial taxa that were significantly over or under-represented in one specific group hence these covariates were not included in subsequent analyses.

**Table 2:** The influence of demographic and clinical characteristics of the FA group on the microbiota

Parameter	Threshold	β diversity (weighted UniFrac)		α diversity (Faith’s PD)
		*q* value	*R*^2^	*q* value
**Age (years)**	<, > 13	0.37	0.006	0.19
**Gender**	F, M	0.29	0	0.26
**Asthma**	Yes, No	0.62	0	0.28
**Eosinophils (median levels)**	<, > 600	0.95	0	0.68
**Eosinophils (extreme levels)**	< 300, > 900	0.94	0.001	0.94
**Adrenaline use**	Yes, No	0.44	0	0.69

### IgE-mediated FA patients have a specific microbial signature that differs from non-allergic

**Fig. 1:**
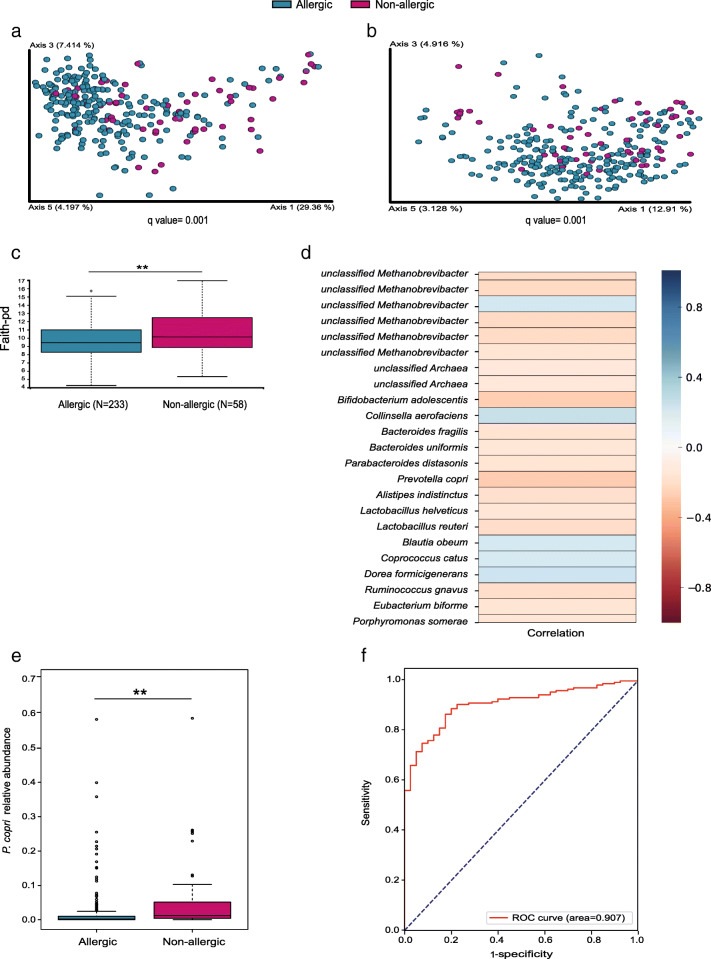
Allergic patients have different gut microbiota compared to healthy non-allergic controls. **a**, **b** PCoA based on **a** weighted and **b** unweighted UniFrac distances (β diversity). **c** α diversity based on Faith’s PD. **d** Heat map of significantly different features comparing FA patients with controls based on a machine learning analysis. **e** Analysis of the composition of microbiomes (ANCOM), followed by pairwise *t* test with FDR correction using R on the relative abundance of *P. copri*. **f** ROC curve analysis using supervised learning for FA classification based on the microbiome data, against true food allergic status. **q ≤ 0.01

We first set out to characterize the gut microbiota of FA patients to understand if they share common characteristics relative to non-allergic controls. When comparing between-sample diversity (β diversity), we found significant differences between all allergic patients and the non-allergic control group (Fig. 1a, b; *q* = 0.001). Notably, the allergic group exhibited significantly lower species richness (α diversity, Fig. 1c, *q* = 0.01). Based on Spearman correlations on log-normalized feature levels and patient classification as healthy (0) or allergic (1), multiple bacterial taxa were significantly related to allergic or non-allergic states (*p* < 0.05). We found 5 species that increased in the allergic group: *Collinsella aerofaciens* (*r* = 0.25), *Dorea formicigenerans* (*r* = 0.22), unclassified *Methanobrevibacter* (*r* = 0.2), *Blautia obeum* (*r* = 0.2), and *Coprococcus catus* (*r* = 0.19). On the other hand, 18 species were increased in the non-allergic group, with *P. copri* (*r* = − 0.27) and *Bifidobacterium adolescentis* (*r* = − 0.26) being the most overrepresented in this group (Fig. 1d, *p* < 0.05). *P. copri* was also found to be the most overrepresented in the non-allergic according to the ANCOM analysis (Fig. 1e, *q* = 0.002). Supervised learning using SVM on the significantly different bacterial features seen in each group facilitated ROC analysis yielding an area under the curve (AUC) of 0.90 in the test set (Fig. 1f). The bacteria in the genera level that were increased in the allergic or non-allergic groups are presented in Additional file 1: Fig. S1. We found the genera *Adlercreutzia* (*r* = 0.34), *Eggerthella* (*r* = 0.33), *Turicibacter* (*r* = 0.33), and the family Erysipelotrichaceae (*r* = 0.27) to be significantly over-represented in the allergic group. In contrast, the genera *Enterococcus* (*r* = − 0.26), *SMB53* (r = − 0.24), *Prevotella* (*r* = − 0.21), and the Enterobacteriaceae family (*r* = − 0.2) were significantly over-represented in the non-allergic group, whereas *Prevotella* was the genus most significantly over-represented in the non-allergic group.

### The allergic state has more impact on the microbiota than the number of allergies

**Fig. 2:**
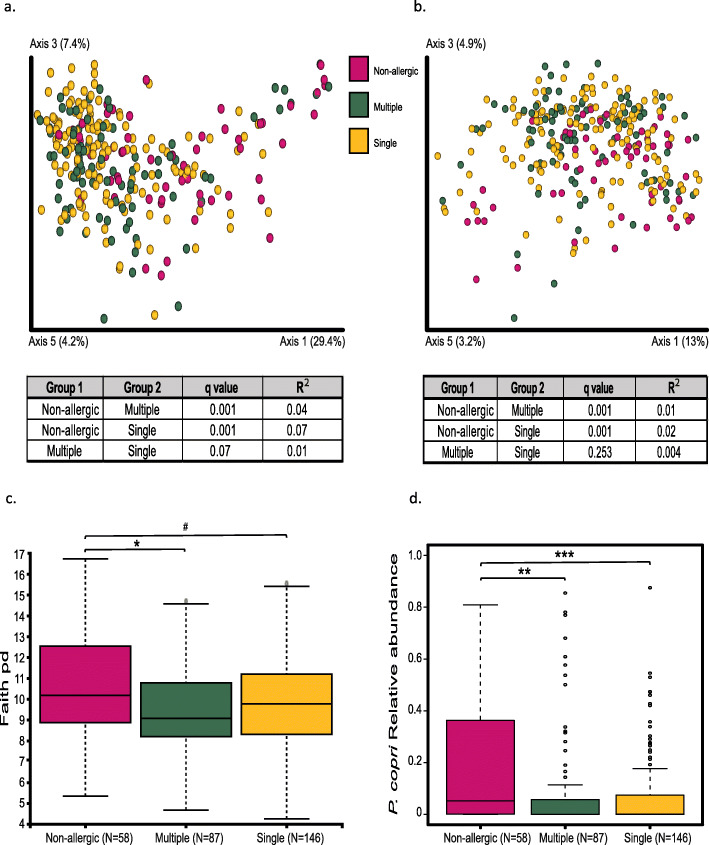
The allergic state has more impact on the microbiota than the number of allergies. **a**, **b** PCoA based on **a** weighted and **b** unweighted UniFrac distances (β diversity). **c** α diversity index based on Faith’s PD. **d** Analysis of composition of microbiomes (ANCOM), followed by pairwise *t* test and FDR correction on the relative abundance of *P. copri*. ^#^*q* < 0.1, *q < 0.05, **q < 0.01, ***q < 0.001

We next divided the 233 allergic patients into two groups: patients with multiple allergies (*n* = 87) and patients with a single allergy (*n* = 146). For this analysis, the tree nuts group single allergy was stringently defined as having either a walnut (pecan and hazelnut included in this group) or a cashew (pistachio included in this group) allergy, but not both (*n* = 20 tree nuts patients excluded). The demographic features of patients with multiple allergies versus single allergy are summarized in Table 3. The two sub cohorts were similar, except for the fact that multiply allergic patients had a significantly higher rate of atopic dermatitis (*p* < 0.001). We characterized the microbial differences between the multiple and the single allergy groups and compared the microbiota to those of non-allergic healthy controls. β diversity demonstrated a significant difference between the non-allergic (*n* = 58) versus the single and the multiple allergy groups, but not between the single and the multiple allergy groups themselves (Fig. 2a,b). Differences were also observed in the α diversity, where the non-allergic group was found to be significantly different from the multiple FA group (*q* = 0.02) and tended to differ from the single FA group (*q* = 0.06), although no significant difference (*q* = 0.26) was found between the single and the multiple allergy groups (Fig. 2c). As above, *P. copri* was the only bacterial feature that differed significantly between the non-allergic group and the multiple and single allergy groups using ANCOM analysis, with no significant differences between the single and the multiple allergies groups (Fig. 2d; *q* = 0.002, *q* = 6e−05, *q* = 0.4, respectively). These results demonstrate that the allergic state, even at the level of a single FA is associated with an altered microbiota, which is not further affected by the multiply-allergic state.

**Table 3:** Demographic characteristics of patients with multiple allergies versus single allergy

Parameter	Single (***n*** = 146)	Multiple (***n*** = 87)	***p*** value
Age, months (IQR)	77.5 (66–121.5)	75 (61–111)	NS
Gender, M (%)	59.31	59.77	NS
BMI *Z*-score median (IQR)*	0.1 (− 0.64–0.84)	0.18 (− 0.43–0.8125)	NS
HDM (%)**	81.58	75.38	NS
AD (%)	17.12	42.53	< 0.001
Asthma (%)	26.90	36.78	NS

*IQR* interquartile range, *BMI* body mass index, *HDM* house dust mite, *AD* atopic dermatitis

*n = 143 and 86 for single allergic and multiply allergic patients, respectively

**n = 114 and 65 single allergic and multiply allergic patients, respectively

### Significant differences in the gut microbiota between the different IgE-mediated FAs

**Fig. 3:**
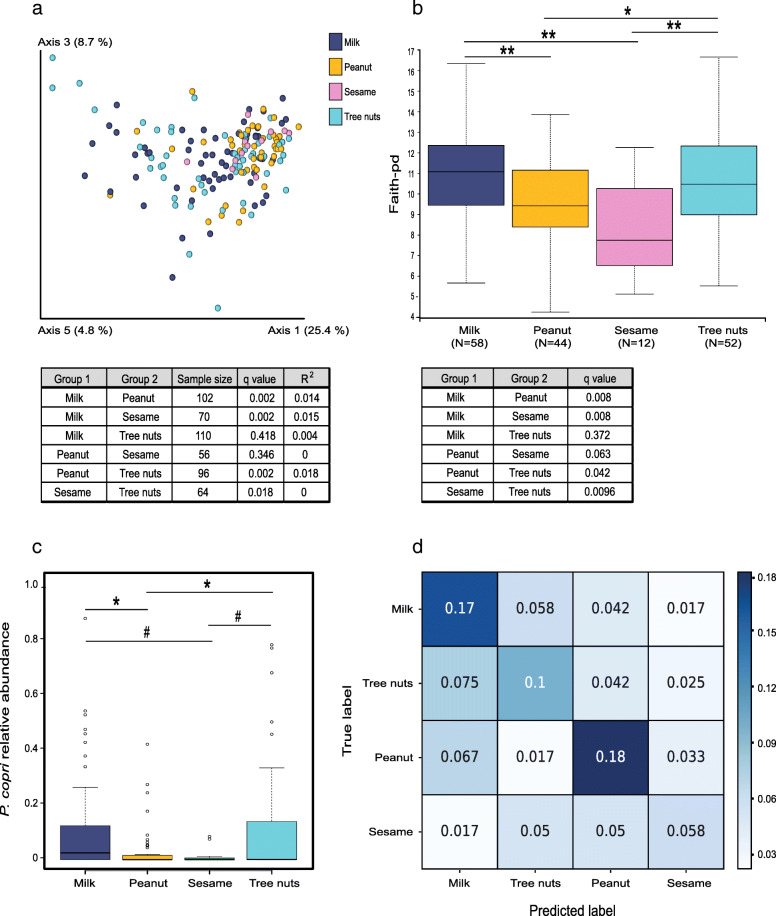
Each allergy type has a distinct gut microbial signature. **a** PCoA based on the weighted UniFrac distances. **b** α diversity index based on Faith’s PD. **c** ANOVA followed by pairwise *t* test and FDR correction on the relative abundance of *P. copri*. **d** Confusion matrix classification for individual allergy groups based on the microbial data using supervised learning. ^#^*q* < 0.1, *q < 0.05, **q ≤ 0.01, ***q < 0.001

**Fig. 4:**
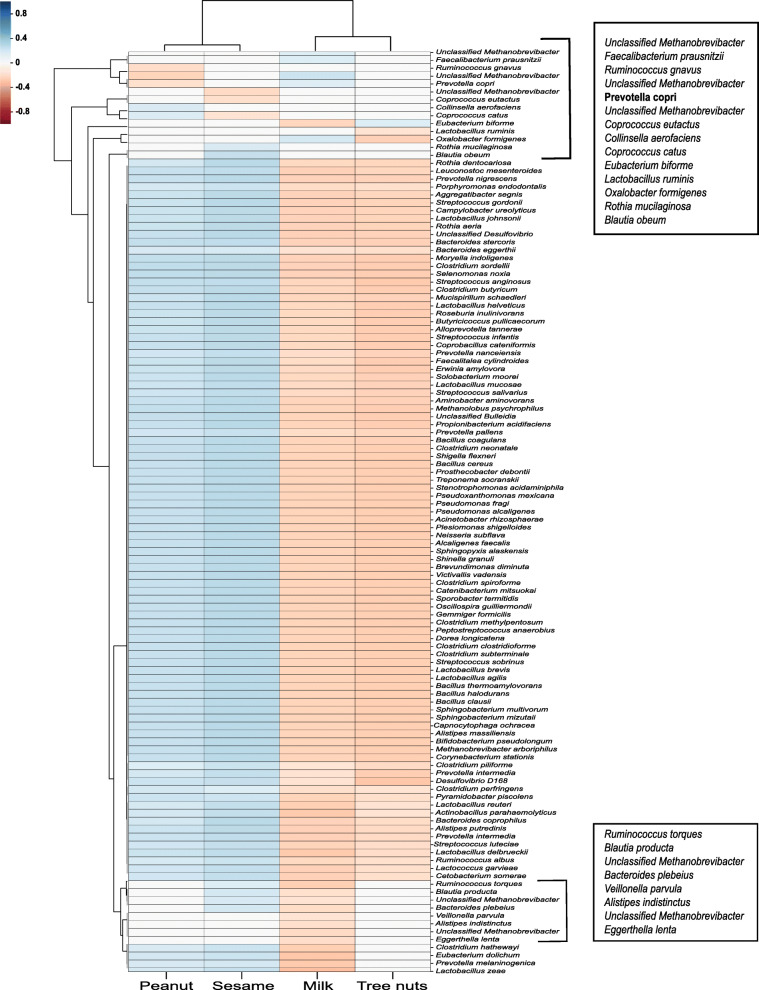
Bacterial features associated with different FAs. Spearman’s correlation between the bacterial features and the classification of the FAs (milk, peanuts, tree nuts, and sesame) among FA patients

In order to characterize the differences in microbiota between subjects with different allergies, we compared patients with only a single FA: milk, peanut, sesame, or tree nuts. In the latter group, patients with a single tree nuts FA typically avoid consumption of multiple tree nuts regardless of the specific nut to which they are allergic and were therefore grouped together. Demographic and clinical features of these groups are presented in Table 4. We found the different IgE-mediated FA to be significantly different in their gut microbiota composition, whereby the β diversity (Fig. 3a) was significantly different in milk versus peanut (*q* = 0.002) and sesame (*q* = 0.002), peanut versus tree nuts (*q* = 0.002), and sesame versus tree nuts (*q* = 0.018). Similar differences were seen in the α diversity, between the same groups, whereas the sesame allergy group had the lowest species richness compared to all of the allergy types examined (Fig. 3b). We performed an ANOVA test for the relative abundance of *P. copri* (*q* = 0.0002) and found significantly higher relative abundance in the milk and tree nuts FAs compared to the peanut allergy (Fig. 3c; *q* = 0.043 for both). We also observed a trend when comparing the relative abundance in the sesame group with the milk and tree nuts groups (*q* = 0.09). Furthermore, using a linear SVM, we tested the classification of four different allergy types. We were able to correctly classify each FA according to the microbiota profile with a total accuracy of 0.51 (51%) in the test set compared with 25% for random classification of 4 classes, demonstrating a unique signature for each allergy type (Fig. 3d). To estimate the accuracy of predicting each FA vs all others, the precision (fraction of true positive instances among all predicted as positive) and recall (sensitivity-the fraction of true positive instances among all truly positive) were computed. Peanut FA prediction was the most accurate with a precision of 0.57 and a recall of 0.61 (compared with 0.25 for random predictions). The precisions and recalls of the classification for milk and tree nuts FA groups were also good ((0.52, 0.59) and (0.44, 0.41) respectively). The accuracy obtained for the sesame FA was low, probably because of the limited sample size (sesame allergic with a single allergy, *N* = 12) (Fig. 3d). Next, in order to find different bacterial features between the different FA, Spearman correlations were computed between the log-normalized bacterial feature levels and the classification of the allergy type (milk, peanut, tree nuts, and sesame) among the allergic population. The correlation was calculated with the flag representing each class (1) versus the others (0) and summarized in Fig. 4. The clustering seen here between milk and tree nuts vs sesame and peanut at the taxonomic levels is consistent with the data from Fig. 3. Furthermore, *P. copri* was found to be negatively correlated with peanut allergy and positively correlated with milk allergy (*r* = − 0.18 and 0.16, respectively). Additionally, *Prevotella* was the only genus differing significantly between the allergy groups in the ANCOM analysis (*W* = 142). Further bacterial positive and negative classifiers correlated with each allergy in comparison to the three other allergy types are presented in Additional file 1: Fig. S2.

**Table 4:** Demographics and clinical characteristics of singly allergic groups

Parameter	Milk (***n*** = 58)	Peanut (***n*** = 44)	Sesame (***n*** = 12)	Treenut (***n*** = 52)	***p*** value
Age, months (IQR)	83 (68.8–133.3)	70 (58.0–86.5)	87 (63.0–118.0)	81.5 (68.3–111.8)	0.009*
Gender, M (%)	49.1	70.4	58.3	63.5	NS
BMI *z*-score mean (IQR)	− 0.22 (− 0.45–0.02)	0.03 (− 0.38–0.43)	− 0.15 (− 0.75–0.44)	0.034 (− 0.03–0.72)	NS
HDM (%)	82.2	74.3	81.8	79.5	NS
AD	8.6	31.8	8.3	26.9	0.01**
Asthma (%)	53.4	34.1	50.0	42.3	NS

*IQR* interquartile range, *BMI* body mass index, *HDM* house dust mite, *AD* atopic dermatitis

*Comparison between milk and peanut *p* = 0.009

**Comparison between milk and peanut *p* = 0.004

### FA patients have significantly lower SCFAs concentrations compared to healthy non-allergic controls

**Fig. 5:**
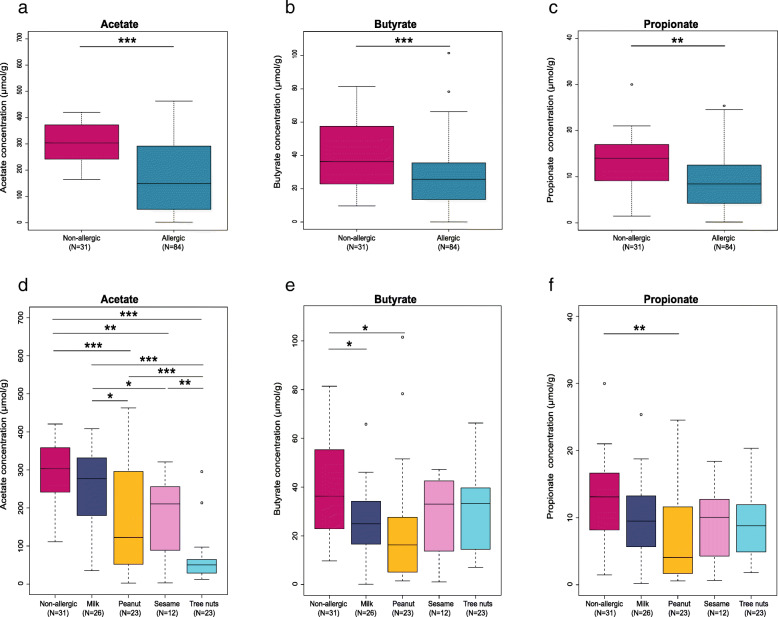
Food allergic patients have significantly lower stool SCFA concentrations compared to non-allergic controls. GC-MS analysis of **a** acetate, **b** butyrate, and **c** propionate concentrations in stool of allergic patients (*N* = 84) compared to the healthy non-allergic control group (*N* = 31). **d** Acetate, **e** butyrate, and **f** propionate concentrations in stool of non-allergic compared to the single FA individually; milk (*N* = 26), peanut (*N* = 23), sesame (*N* = 12), and tree nuts (*N* = 23) by ANOVA followed by pairwise *t* test and FDR correction. *q < 0.05, **q ≤ 0.01, ***q ≤ 0.001

We evaluated the SCFAs concentrations in the stool samples of 84 singly allergic patients and compared them to 31 age-matched non-allergic healthy controls from our cohort. We found significant differences between the SCFAs concentrations in allergic patients compared to the non-allergic group. Acetate, butyrate, and propionate concentrations were each significantly lower in the group of the allergic patients compared to the non-allergic group (Fig. 5a–c; *q* = 1.6e−08, *q* = 0.001, *q* = 0.002, respectively). When comparing the SCFAs concentration between the non-allergic control group and the single allergy groups (milk *N* = 26, peanut *N* = 23, sesame *N* = 12, tree nuts *N* = 23) (Fig. 5d–f), we found multiple differences in the acetate concentrations (Fig. 5d) between the non-allergic group and the peanut, sesame, and tree nuts groups (*q* = 0.0008, *q* = 0.002, *q* = 1.1e−11, respectively). Moreover, significant differences were found between the FA groups themselves, namely milk vs. peanut, sesame, and tree nuts (*q* = 0.02, *q* = 0.04, *q* = 6.7e−08, respectively), and tree nuts vs. sesame and peanut (*q* = 0.008, *q* = 0.001, respectively). Additionally, significant differences were found in the butyrate concentration between the non-allergic vs. the milk and peanut groups (Fig. 5e; *q* = 0.02) and in the propionate concentration between the non-allergic and the peanut group (Fig. 5f, *q* = 0.009).

### Correlations of bacterial features with SCFA concentrations

**Fig. 6:**
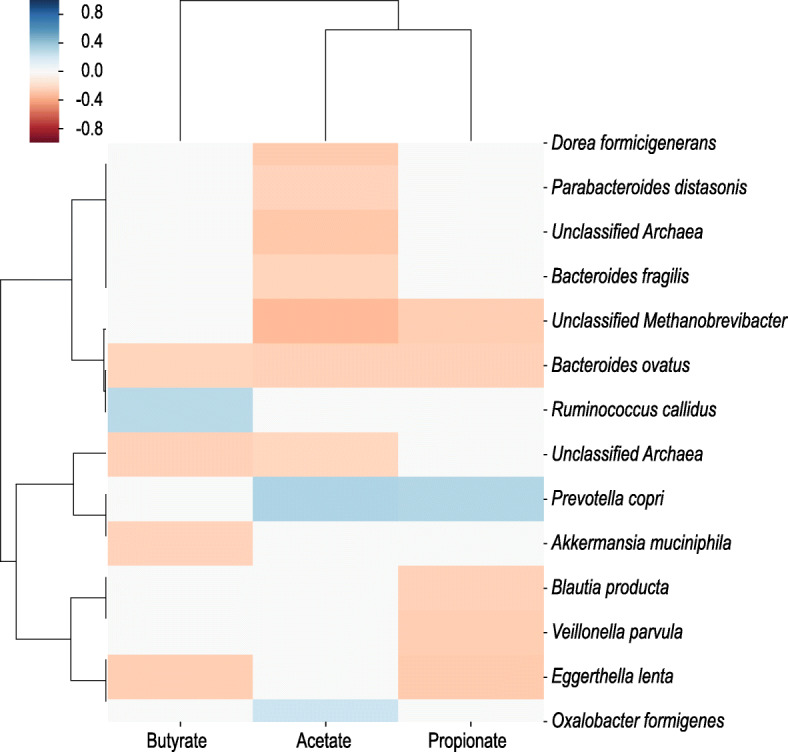
Correlation between the SCFAs and the bacteria of FA. Spearman correlations between bacterial features and levels of SCFAs (acetate, butyrate, and propionate) among FA patients

Given the potential relevance of SCFAs in modulating FA, we performed Spearman correlations between the continuous values of the SCFAs (acetate, butyrate, and propionate) and the bacterial features from the same FA-derived samples. The results are summarized in a heatmap (Fig. 6, *p* = 0.05). We found *P*. *copri* to have a significant positive correlation with acetate and propionate in the allergic patients (*r* = 0.33, 0.32, respectively). In addition, *Oxalobacter formigenes* was positively correlated with acetate (*r* = 0.23) and *Ruminococcus callidus* was positively correlated with butyrate (*r* = 0.29) (Fig. 6).

### FA patients are predicted to have significantly fewer acetate-related functional pathways

**Fig. 7:**
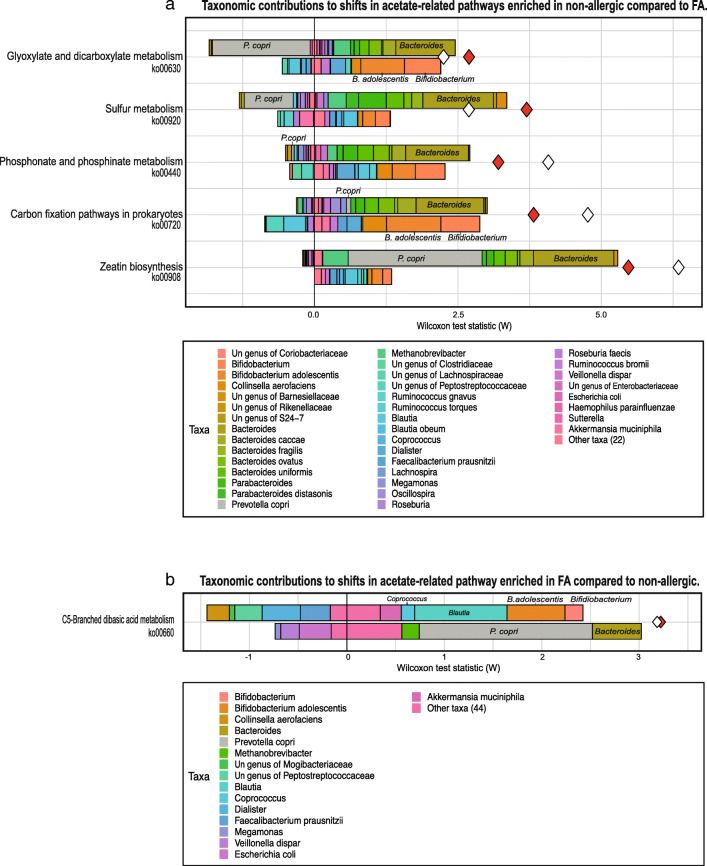
Acetate pathways differ between allergic and non-allergic individuals. **a** Taxonomic contributors of differentially abundant acetate-related KEGG pathways, as quantified by FishTaco (only those with FDR < 0.05 are shown). The taxonomic contributors are separated into taxa that are over-represented in the group the pathways are enriched in and contribute positively to the observed shift in cases (upper right bars), taxa that are over-represented in the group the pathways are depleted in and contribute positively to the observed shift in cases (lower right bars), and taxa that are attenuating the observed shift (left bars). Taxa contributing to the observed over-representation of acetate-related KEGG pathways in the non-allergic group compared to the FA group. **b** Taxa contributing to the observed over-representation of an acetate-related KEGG pathway in the FA group compared to the non-allergic group. The red diamonds represent the Wilcoxon rank-sum statistic (*W* statistic) for the difference in pathway abundances between groups inferred from KO profiles, and the white diamond represents the *W* statistic when comparing pathway abundances between groups inferred from taxonomic profiles and genomic content. Un, unclassified

**Fig. 8:**
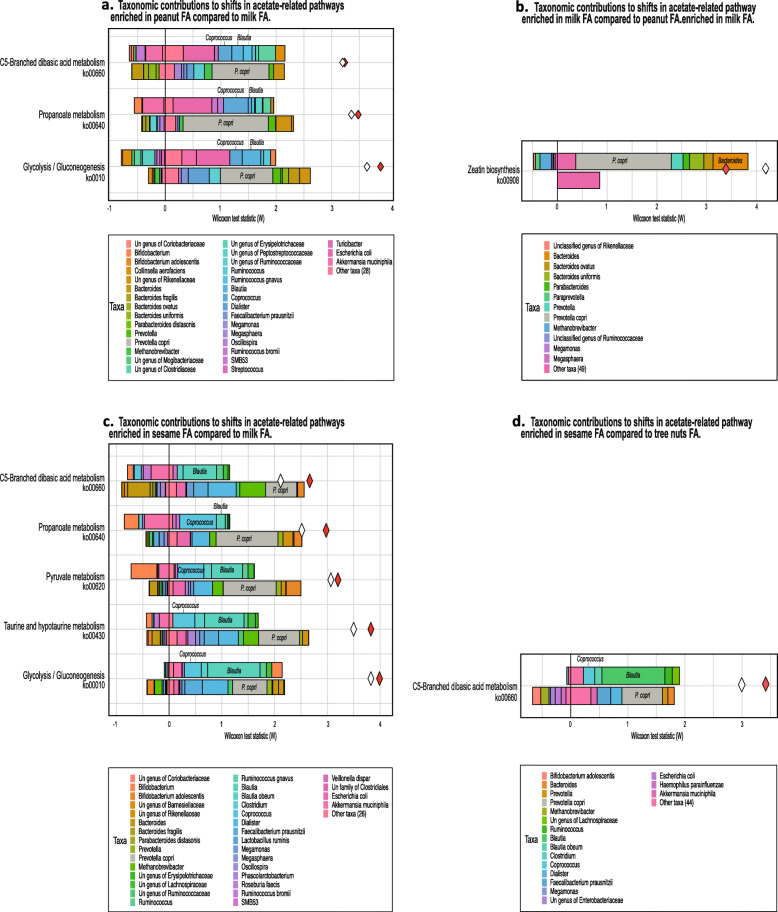
Acetate pathways differ between the FAs. Taxonomic contributors of differentially abundant KEGG pathways between the different FA groups, as quantified by FishTaco (only those with FDR < 0.05 are shown). **a** Taxa contributing to the observed over-representation of several KEGG pathways in the peanut FA group compared to the milk FA group. **b** Taxa contributing to the observed over-representation of several KEGG pathways in the milk FA group compared to the peanut FA group. **c** Taxa contributing to the observed over-representation of several KEGG pathways in the sesame FA group compared to the milk FA group. **d** Taxa contributing to the observed over-representation of several KEGG pathways in the sesame FA group compared to the tree nuts FA group. Un, unclassified

Finally, we sought to identify the specific pathways that differ between the allergic and non-allergic group. FishTaco analysis of PICRUSt2-predicted functional profiles has identified 52 KEGG pathways that were significantly enriched in the non-allergic group, compared to 21 pathways significantly enriched in the FA group (Additional file 2: Table S1, FDR < 0.05). More specifically, we found 5 acetate-related KEGG pathways significantly enriched in the non-allergic group (Fig. 7a, i.e., ko00908, ko00720, ko00440, ko00920, ko00630) compared to one that was enriched in the allergic group (Fig. 7b, ko00660). Moreover, FishTaco analysis has indicated that the functional shifts of the enriched acetate-related pathways in the non-allergic group were mainly driven by *Bacteroides*. In addition, this analysis indicated that *P*. *copri* had a major contribution to the shift in one functional pathway (zeatin biosynthesis) but attenuated the shift in two other functional pathways (sulfur metabolism and glyoxylate and dicarboxylate metabolism) (Fig. 7a). We also found that the functional shifts of the enriched acetate-related pathway in the allergic, C5-branched dibasic acid metabolism, was driven by increased abundance of *Blautia* and by decreased abundance of *P. copri* in the FA group (Fig. 7b). The full list of taxonomic drivers of differences in acetate-related pathways between allergic and non-allergic are summarized in Additional file 2: Tables S2, S3. In addition, we found several functional dissimilarities and differences in acetate-related pathways between the different FAs (Fig. 8), with *P. copri*, *Bacteroides*, *Coprococcus*, and *Blautia* being the key contributors to observed functional shifts between the various FAs (Fig. 8a–d; and see Additional file 2: Tables S4–S7 for details).

## Discussion

The development and persistence of FA involves several biological systems and their integrated responses to ingested substances. The location and scope of the human gut microbiota uniquely situate it to exert influence on the progression and establishment of FA. In this study comprised of 233 FA patients and 58 non-allergic healthy controls, we characterize a gut microbiota signature and SCFA profiles of patients with persistent FA to tree nuts, peanuts, milk, or sesame, compared to non-allergic individuals. We demonstrate that there is a distinct microbial signature in FA, with distinguishing features among the different FA. FA-associated gut microbial composition is significantly different from the non-allergic composition, and FA patient stool contains significantly lower concentrations of SCFAs compared to that of non-allergic individuals. Thus, our study suggests the presence of FA-associated microbiota signatures and a possible functional mechanism linking microbiota composition to allergy.

FA arises on a background of Th1-Th2 imbalance, resulting in the Th2 differentiation of allergen-specific T cells, IgE-switching of B cells, and mediator release of effector cells such as basophils and mast cells. Negating these effects, components of the gut microbiota can promote oral tolerance in several ways. Recognition of gut microbiota components by toll-like receptors (TLR) on resident dendritic cells drives regulatory T cell (Treg) generation in a TGFβ-dependent manner [31]. SCFAs produced by microbiota fermentation are recognized by G protein-like receptors on intestinal epithelium which leads to improved intestinal homeostasis and barrier function [32]. SCFA also can directly influence Treg development [3] and IL10 expression in Th1 cells [33], both of which through T cell expression of GPR43. Additionally, the commensal microbiota also serve to protect against colonization by opportunistic species, for example by controlling the availability of nutrients [34, 35] or stimulating host production of antimicrobial peptides [36, 37]. In contrast, a shift in the microbiome composition will often manifest with microbial populations that do not promote the above protective effects [13, 38], thereby facilitating the development of Th2 immune orientation.

Our findings point to altered features within the microbiota signatures between allergic and age-matched non-allergic healthy controls, namely a significantly different microbiota composition and lower species richness, which suggests an altered gut microbiota in the FA state. Although microbiota richness and diversity can be reflective of underlying clinical or demographic characteristics of study subjects, grouping by these factors (specifically, gender, age, asthma, and eosinophilia) did not yield microbial differences in contrast to grouping by allergic or non-allergic state. This strong effect allowed to distinguish the allergic from the non-allergic individuals reflected by a 90% AUC using supervised ML based on the bacterial features in each group.

We found *P*. *copri* to be the most significantly over-represented species in the non-allergic group compared to the FA group. This observation fits with a recent Australian prebirth case-cohort study, demonstrating that maternal carriage of *P. copri* during pregnancy, strongly predicted the absence of food allergy in the offspring [39] which again strengthens our observation that higher abundance of *P. copri* is negatively associated with FA. SCFAs levels were also lower in the allergic group and specifically acetate, which may be related to the dearth of microbial species such as *P*. *copri* which synthesize acetate and propionate as metabolites from digestion of complex carbohydrates. Not surprisingly, the species we found correlated with SCFAs concentration in FA-stool were also reported to associate with SCFAs production in the gut, i.e., *P. copri* which produces acetate and propionate and *R. callidus* which is a known acetate producer. The correlation of *P. copri* with acetate was not observed in the non-allergic controls; however, despite higher levels compared to FA patients. This may be reflective of the multiple acetate-related KEGG pathways enriched in the controls, most of which were associated with *Bacteroide*s, with one (zeatin biosynthesis) being strongly associated with *P. copri*. Overall, these findings emphasize the potential multifaceted roles of the gut microbiota in driving metabolic pathways that are protective against FA development.

The higher relative abundance of *P. copri* in the stools of the non-allergic control group compared to the group with FA may partially explain the concomitant increase in SCFAs, in particular propionate, which depends on a dietary intake rich in fiber and complex carbohydrates [40]. It has in fact been shown that *P. copri* is predominantly found in stool of non-Western populations whose diets tend to be enriched for these components [41, 42]. In addition, *P. copri* is able to metabolize dietary metabolites to produce succinate [43], a carboxylic acid that stimulates innate immune cell development. At the same time, *P. copri* has also been associated with increased incidence of Th1 and Th17 inflammatory pathologies such as colitis and rheumatoid arthritis (RA) [44, 45], with direct Th1 T cell responses to *P. copri* reported in patients with RA [46]. This may suggest that the characterization of specific species as beneficial or harmful could depend on context, including that of other background microbiota species.

The cause for different relative abundance of *P. copri* in the different FAs is not fully understood and might be explained by a variety of known allergen-related risk factors: genetic, environmental, or dietary habits, such as lack of breastfeeding and a low-fiber diet [47]. Further research needs to be carried out in order to elucidate the contribution of different factors to the different abundances of *P. copri* in FAs and its effects on the FA disease state.

This study has several limitations. Regarding nutritional inputs, it is well appreciated that the dietary intake can tailor the development of particular microbiota populations. While we cannot categorically claim that the microbiota differences observed between the different FA groups were not influenced by nutritional differences, the overall differences in microbiota composition between FA patients and healthy controls (which can be segregated as indicated by ROC analysis) are indicative of a microbiome influenced by the FA state. Furthermore, we would expect that the dietary restrictions for singly allergic tree nuts or peanut patients would have only a limited effect on overall nutrition, albeit for milk allergy the effect may be more comprehensive [48, 49]. In addition, as patients with milk allergy and those with tree nuts allergy have completely different diets, we expected to see major differences in their microbiota, but in fact, we observed the opposite results. The milk and tree nuts FA groups exhibited similarities in their microbiota characteristics (Fig. 4) further strengthening the association of the mere presence of FA with a microbial signature rather than different diets. Despite this, BMIs were not significantly different between the tree nuts, peanut, and milk allergic groups. Regarding our FA patient cohort, this study evaluated those who, due to their age, were likely persistently allergic. As such, our study should not necessarily be taken to describe the etiology of FA. Finally, while we report on associations between specific microbiota and FA, a direct role for identified microbiota and their metabolites remains to be investigated.

Although the microbiome in FA is a widely researched subject, there is still much to be understood. Microbiota differences have been observed in patients with FA, and they differ based on the food allergen studied. Fazolollahi et al. reported a higher prevalence of Lachnospiraceae, Streptococcaceae, and Leuconostocaceae in children with egg allergy [19] and Berni et al. reported an increase in Lachnospiraceae and Ruminocaceae in those with milk allergy [50]. One study addressed the subject of milk allergy resolution and found that the presence of Clostridia and Firmicutes in the gut was associated with resolution of milk allergy by 8 years of age [16]. However, these studies remained mostly at the higher taxonomical levels such as phylum, class, or family level at most. Thus, there is a need to identify specific bacterial species, in order to develop a potential treatment for FA. Our study provides a higher bacterial resolution discovering specific missing species in allergic patients, also correlating to SCFAs levels found in the stool. This might potentially provide guidance for FA-related beneficial species that are candidates for further research. In addition, we have discovered different KEGG pathways enriched in non-allergic individuals and reduced in allergic individuals and the taxonomic contributors to each differentially abundant pathway, thus providing an insight towards a possible mechanistic link between these bacteria and the development of FA. Moreover, we provide additional information with the use of ML, presenting high accuracy for FA identification based on the microbiota information alone. Finally, we demonstrate differences between particular single FAs, with a comparison to the multiply allergic state.

## Conclusions

In summary, in this large study of patients with FA, we demonstrate microbiota population characteristic of the general persistent FA state, including lower alpha diversity and lower abundance of SCFA-producing bacteria. This was associated with decreased SCFA concentrations in stool and also significantly fewer acetate-related pathways, thus suggesting that the dearth of SCFAs may have a role in causing the allergic state. Furthermore, each FA manifests distinctive microbiota populations. Utilizing ML, different allergic states could be classified by microbiota differences, validating our findings, and highlighting their potential diagnostic use following further optimization. Future work will address the roles played by microbiota in the food allergic state and the potential for probiotic and postbiotics therapies in ameliorating allergic disease.

## Supplementary information

**Additional file 1: Figure S1.** Allergic patients have specific significant bacteria. Bacteria classified by higher taxonomic order (genus level), associating with the allergic or non-allergic groups. **Figure S2.** Each allergy type has specific significant bacteria.

**Additional file 2: Table S1.** Differential abundance statistics of KEGG pathways in FA and non-allergic; **Table S2.** Taxonomic contributions to shifts in acetate-related pathways enriched in non-allergic compared to FA; **Table S3.** Taxonomic contributions to shifts in acetate-related pathway enriched in FA compared to non-allergic; **Table S4.** Taxonomic contributions to shifts in acetate-related pathways enriched in peanut FA compared to milk FA; **Table S5.** Taxonomic contributions to shifts in acetate-related pathway enriched in milk FA compared to peanut FA; **Table S6.** Taxonomic contributions to shifts in acetate-related pathways enriched in sesame FA compared to milk FA; **Table S7.** Taxonomic contributions to shifts in acetate-related pathway enriched in sesame FA compared to tree nuts FA.

## Data Availability

The datasets generated during and analyzed during the current study are available in the European Nucleotide Archive repository with the accession number PRJEB37877 (https://www.ebi.ac.uk/ena/browser/view/PRJEB37877) [51].
