# Supplementary material for: Microbial signature in IgE-mediated food allergies
Source: Genome Med. 2020 Oct 27;12:92. doi: 10.1186/s13073-020-00789-4 (PMC7592384; doi:10.1186/s13073-020-00789-4)
Supplement: Supplementary file 1 — Additional file 1: Figure S1. Allergic patients have specific significant bacteria. Bacteria classified by higher taxonomic order (genus level), associating with the allergic or non-allergic groups. Figure S2. Each allergy type has specific significant bacteria. [file 13073_2020_789_MOESM1_ESM.docx]

**Fig S1. Allergic patients have specific significant bacteria.** Bacteria classified by higher taxonomic order (genus level), associating with the allergic or non-allergic groups.

**
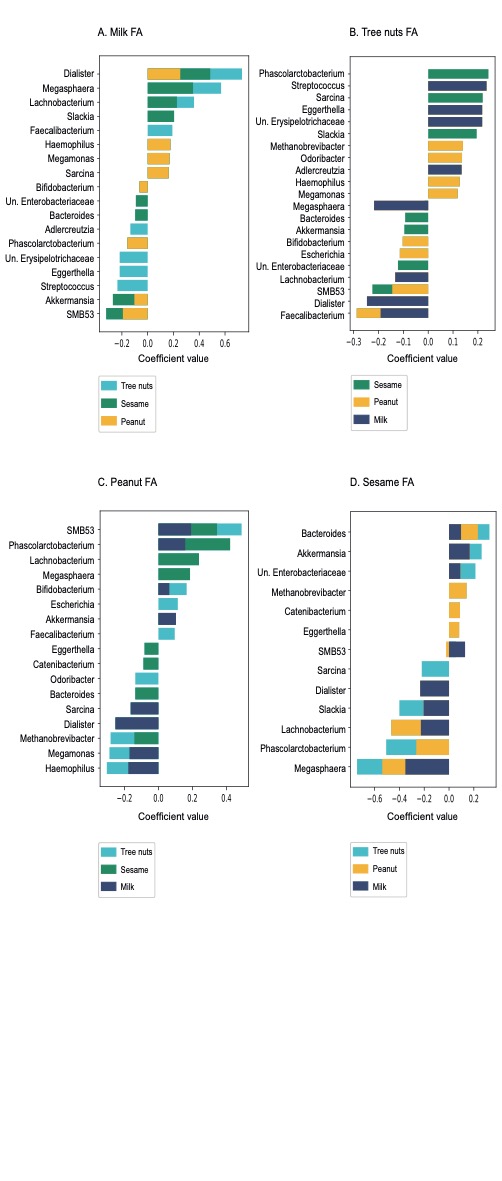
**

**Fig S2. Each allergy type has specific significant bacteria.** (A-D) Bacteria classified by higher taxonomic order (genus level), associating with each allergy in comparison to the three other allergy types. (A) milk FA, (B) tree nuts FA, (C) peanut FA, (D) sesame FA.
